# The Impact of Green Tea Kombucha on the Intestinal Health, Gut Microbiota, and Serum Metabolome of Individuals with Excess Body Weight in a Weight Loss Intervention: A Randomized Controlled Trial

**DOI:** 10.3390/foods13223635

**Published:** 2024-11-14

**Authors:** Gabriela Macedo Fraiz, Dandara Baia Bonifácio, Udielle Vermelho Lacerda, Rodrigo Rezende Cardoso, Viviana Corich, Alessio Giacomini, Hércia Stampini Duarte Martino, Sergio Esteban-Echeverría, Ana Romo-Hualde, David Muñoz-Prieto, Frederico Augusto Ribeiro de Barros, Fermín I. Milagro, Josefina Bressan

**Affiliations:** 1 Department of Nutrition and Health, Universidade Federal de Viçosa, Viçosa 36570-900, Brazil; gabriela.fraiz@ufv.br (G.M.F.); dandara.bonifacio@ufv.br (D.B.B.); hercia@ufv.br (H.S.D.M.); jbrm@ufv.br (J.B.); 2 Department of Nutrition, Food Science and Physiology, Centre for Nutrition Research, Universidad de Navarra, 31008 Pamplona, Spain; sergioestebanecheverria@gmail.com (S.E.-E.); aromo@unav.es (A.R.-H.); dmunozp@unav.es (D.M.-P.); 3 Department of Food and Technology, Universidade Federal de Viçosa, Viçosa 36570-900, Brazil; udielle.lacerda@ufv.br (U.V.L.); rodrigocardoso@ufv.br (R.R.C.); fredbarros@ufv.br (F.A.R.d.B.); 4 Department of Agronomy, Food Natural Resources, and Environment (DAFNAE), Università degli Studi di Padova, 35020 Padova, Italy; viviana.corich@unipd.it (V.C.); alessio.giacomini@unipd.it (A.G.); 5 Centro de Investigación Biomédica en Red de la Fisiopatología de la Obesidad y Nutrición (CIBERobn), Institute of Health Carlos III, 28029 Madrid, Spain; 6 Navarra Institute for Health Research (IdiSNA), 31008 Pamplona, Spain

**Keywords:** fermented food, gastrointestinal microbial community, intestinal permeability, metabolomics, obesity

## Abstract

Green tea kombucha (GTK) has emerged as a promising probiotic fermented beverage. Few studies have investigated its effect on human health, mainly focusing on intestinal health, microbiota composition, and metabolomics. The present study is a pioneer in investigating the effect of GTK consumption in individuals with excess body weight. This is a randomized controlled trial, lasting ten weeks, with two groups placed under an energy-restricted diet: control (CG, *n* = 29), kombucha (KG, *n* = 30; 200 mL/d). Biological samples and questionnaires were collected before and after the intervention. Microbiota analysis used an amplification of the V4 region of 16S rRNA. Serum untargeted metabolomics used HPLC-TOF mass spectrometry. Intestinal permeability considered the urine excretion of lactulose and mannitol, plasma zonulin, and LPS-binding protein. After the intervention, no differences related to intestinal permeability and microbiota were found between groups, but only the CG had increased fecal pH, lactulose/mannitol ratio, and zonulin. In addition to this, the KG reported lower gastrointestinal symptoms related to motility compared to the CG, and discriminant metabolites (e.g., diethyl malonate) were found strictly in the KG. GTK did not significantly improve gut microbiota and intestinal permeability. However, GTK ameliorated gastrointestinal symptoms and positively influenced the serum metabolome, which may contribute to enhancing the metabolic health of individuals with excess body weight.

## 1. Introduction

Fermented foods, defined as “foods made through desired microbial growth and enzymatic conversions” by the International Scientific Association for Probiotics and Prebiotics, have gained notable popularity in recent years [1]. Kombucha, a fermented tea beverage, has emerged as one of these foods, with promising therapeutic benefits that could enhance intestinal health by modulating microbiota, combating metabolic imbalance, and acting as an antioxidant agent [2]. Kombucha is usually made with the infusion of sugared green or black (Camellia sinensis) tea, fermented by a symbiotic culture of bacteria and yeasts (SCOBY). The processes that occurs during the fermentation results in a final composition rich in bioactive compounds, such as phenolic compounds, organic acids, enzymes, bioactive peptides, vitamins, and minerals [3].

Despite the increasing commercial expansion and consumption of kombucha, little is known about its real effects on human health, especially in individuals with overweight and obesity [4]. Excess body weight leads to metabolic changes that worsen the body’s overall homeostasis, promoting low-grade inflammation and negatively impacting intestinal microbiota [5]. Individuals with excess body weight present a different profile of gut microbiota composition, in addition to lower diversity and richness, often implicated in the pathogenesis of metabolic disorders [6]. Simultaneously, there is a weakening in the integrity of the intestinal barrier accompanied by bacterial endotoxins trespassing into the bloodstream, leading to endotoxemia and enhancing low-grade systemic inflammation [7].

In this manner, understanding the interplay between targeted food strategies, such as kombucha consumption, and intestinal health holds promise for elucidating the impact of this beverage on human health, mainly in the context of obesity [8]. Studies in animal models suggest that green tea kombucha may help prevent obesity by reducing gut dysbiosis, supporting intestinal barrier function to limit endotoxin translocation and reduce adipose tissue inflammation [9,10]. Moreover, exploring host metabolite profiles through untargeted serum metabolomics is also of great interest since the components of functional foods, such as phenolic compounds, can serve as substrates to the metabolism of microbiota [11].

To our knowledge, there are no studies that have investigated the effect of green tea kombucha consumption in the context of an energy-restricted diet on the intestinal parameters and serum metabolome in human health. Therefore, this study aims to bridge this gap by examining the influence of beverage intake on the gastrointestinal symptoms, intestinal permeability, microbiota diversity and composition, and serum metabolites of individuals with excess body weight in the context of a weight loss treatment.

## 2. Materials and Methods

### 2.1. Experimental Design and Participants

This is a parallel randomized controlled clinical trial with a duration of 10 weeks that included two groups of individuals with excess body weight: (1) control (CG), under an energy-restricted healthy diet and (2) kombucha (KG), with the same diet plus 200 mL/day of green tea kombucha (GTK) [12]. All participants had a Body Mass Index (BMI) ≥ 27 kg/m^2^, waist circumference (WC) ≥ 80 cm for women and ≥94 cm for men, and body fat (BF) > 30% for women and >25% for men [13]. Individuals diagnosed with metabolic or chronic diseases, those regularly using medications, nutritional supplements, and with a habitual consumption of teas and/or fermented foods were excluded from the study [12]. Eating behaviors were evaluated at the screening phase to not include individuals with disruptive habits that might impact the intervention. This assessment used the 21-item Three-Factor Eating Behavior Questionnaire tailored for the Brazilian population [14].

Following screening, a run-in period of seven days was had to identify participants unlikely to adhere to the research protocol. During this period, participants were instructed to keep their usual physical activity pattern and dietary habits, avoiding nuts, fermented foods, olive oil, teas, and alcohol. The use of nutritional supplements, antibiotics, anti-inflammatory drugs, and smoking were also restricted. They were required to maintain their weight within a 2% variation of their starting weight. Participants who did not follow these guidelines were excluded.

Allocation was performed after the run-in period through the stratified minimization method based on BMI, age, and sex, using the MinimPy software, version 2.0 (Copyright © 2024 Python Software Foundation; https://pypi.org/project/MinimPy/, accessed on 5 March 2023) to ensure a balanced distribution of potential factors that could interfere with the outcome variables. The sample size was determined using G*Power 3.1 software [15], considering an effect size of 0.7 between changes in BF (kg) [16], with a significance level of 5% and a power of 80%. It resulted in a minimum of 31 participants per group, after accounting for a potential 20% dropout rate; the final required number was adjusted to 37 per group [12].

**Figure 1:**
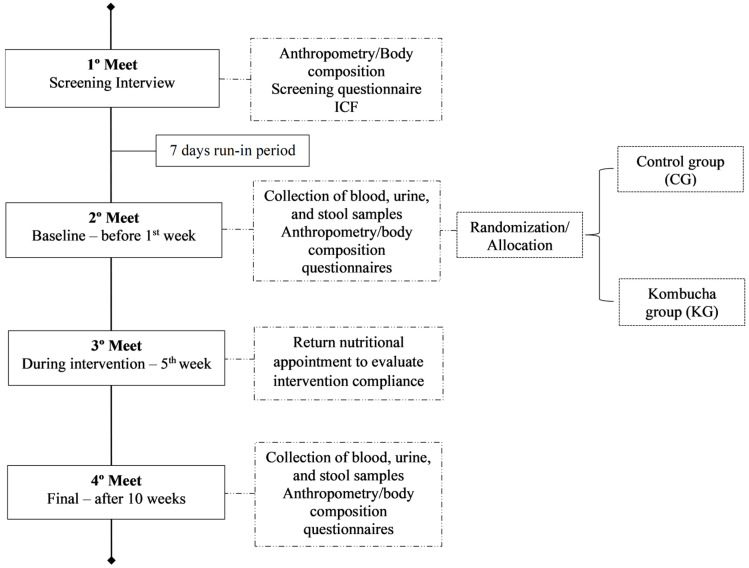
Experimental design. This is a randomized controlled trial involving individuals with excess body weight allocated in control or kombucha groups. All participants attended the first meeting for screening; those who met the inclusion criteria had to accomplish a run-in period. Participants went to a second meeting to collect all the data and biological samples. In the middle of the intervention, after 5 weeks, they had a nutritional return appointment. After 10 weeks, all participants repeated the data collection. ICF: Informed Consent Form.

During the intervention, the individuals were not able to change the usual pattern of physical activity. It was measured using the International Physical Activity Questionnaire validated for the Brazilian population [17]. All lifestyle and health questionnaires, anthropometry/body composition, and the collection of biological samples were assessed at the baseline and end of intervention to compare the data from these two moments (Figure 1).

### 2.2. Ethical Aspects

The Human Research Ethics Committee of the Federal University of Viçosa (UFV) approved this project (CAAE: 25880819.3.0000.5153; number: 6.197.412; date: 24 February 2023) and it was also registered in the Brazilian Registry of Clinical Trials (REBEC) (number: RBR-9832wsx). All procedures outlined were conducted in accordance with CNS Resolution 466/2012 and the Declaration of Helsinki. Participants who consented to join the study signed the informed consent form prior to the intervention. The benefits and risks associated with the research were fully explained, including the assurance that they could withdraw from the study at any point without any negative consequences. Participant identities were kept confidential.

### 2.3. Sample Collection

Participants received instructions to collect their feces in a sterile container (50 mL) within 24 h before the day of appointment and to transport it frozen inside an icebox to the Laboratory of Energy Metabolism and Body Composition (LAMECC). They were also directed to hand in the total volume of fasting urine collected in a 2 L receptacle from the period after dinner the day before the exam until the moment of arrival at the laboratory. On the same day that they brought these biological materials, they stayed at LAMECC for an intestinal permeability test. Individuals underwent an 8 h fasting period prior to sample collection.

On a second day, blood collection was performed at the Clinical Analysis Laboratory of the Health Division—UFV (LACDSA) after an overnight fast of 10 h. It was collected in vacuum EDTA tubes and clot-activator gel for serology tubes for sequent centrifugation at 3500 rpm for 10 min (4 °C) for serum and plasma separation. All the urine, fecal, plasma, and serum samples were aliquoted and stored at −80 °C (Thermo Fisher Scientific, Waltham, MA, USA/Forma 900 Series^®^) until further analysis.

### 2.4. Dietary Intervention

Participants in both groups received a food plan that included five different menus formulated using Brazilian food tables [18]. Each food plan was calculated with a 500 kcal reduction from their daily caloric requirement estimated using the Estimated Energy Requirement—EER [19]. The macronutrient distribution consisted of 55% carbohydrates, 30% lipids, and 15% proteins, following obesity management guidelines [20]. Participants were advised not to modify the food or meals between the menus. Additionally, they received qualitative nutritional guidance on the model of a healthy plate, levels of food processing, and tips on making better food choices, including shopping advice. A document containing all the prescribed recipes was also provided to them.

Trained nutritionists evaluated food consumption through Food Frequency Questionnaires—FFQs—that quantified an individual’s usual food intake before and after the intervention, focusing on their intake over the preceding 10 weeks of study. Food data were converted into grams and milliliters through the ERICA-REC24h software version 24_05_2022 (Copyright © 2011 Projeto Erica). For any foods or preparations not included in this database, the Brazilian Table of Food Composition—the TBCA—was used [21].

### 2.5. Kombucha Production and Dosage

The GTK was produced in the Cereal Chemistry and Technology Laboratory in the Department of Food Technology, UFV, and is described elsewhere [12]. In summary, it used 12 g/L of green tea leaves (Helio Amaya Cia Ltd., Registro, SP, Brazil), 50 g/L of crystal sugar, and 3% of the symbiotic culture of bacteria and yeasts (SCOBY) (*w*/*v*) (Enziquímica Produtos Químicos Ltd., Gravataí, RS, Brazil). A previously fermented kombucha was incorporated into the beverage to lower the pH to 4.2–4.4 to inhibit the growth of pathogenic microorganisms. The fermentation process lasted 5 days, with a controlled temperature of 25 °C. GTK was produced weekly and individuals of the KG consumed 1 bottle per day (200 mL/d), preferably at lunch or with food to avoid the lower gastric pH from reducing the bioavailability of bacteria and yeast presented in the beverage. Individuals from the KG had to fill out daily a printed diary indicating the day and time of ingestion of the drink; those who discontinued kombucha intake for three consecutive days were excluded from the intervention. The kombucha’s characterization is detailed in Fraiz et al. (2024) [12]. The dosage of 200 mL/d of GTK was chosen to provide the necessary amount of Colony-Forming Units (CFUs) to be considered a probiotic drink (>1 billion CFUs per serving). The daily intake of GTK provided 3.96 × 10^9^ of lactic acid bacteria, 2.14 × 10^9^ of acetic acid bacteria, and 3.14 × 10^9^ of yeasts.

### 2.6. Quality of Life and Gastrointestinal Questionnaires

The participant’s quality of life was assessed through the Brazilian-Portuguese version of the 36-Item Short Form Health Survey (SF-36), a self-administered survey that provides information on an individual’s health status [22].

Differences related to gastrointestinal symptoms at the beginning and end of the study were evaluated through the translated and Brazilian validated Gastrointestinal Symptom Rating Scale (GSRS) [23]. It is a self-completed questionnaire consisting of 15 questions across 5 domains: diarrhea, constipation, bloating, abdominal pain, reflux, and indigestion. The questionnaire answers used the 7-point Likert scale, in which “1” represents absence and “7” is the most frequent or intense symptom.

The Bristol Stool Scale (BSS) was also used to analyze the stool consistency and form. There are 7 classifications of the BSS: (1) hard lumps resembling nuts; (2) lumpy, sausage-shaped stools; (3) sausage- or snake-like with cracks on the surface; (4) smooth and soft, sausage- or snake-like stools; (5) soft pieces with well-defined edges; (6) fluffy, ragged-edged pieces, forming a mushy stool; and (7) liquid with no solid fragments [24].

### 2.7. Intestinal Permeability, Fecal pH, and Short-Chain Fatty Acids (SCFA)

Intestinal permeability was assessed through the administration of non-metabolizable substances of different molecular weights that were quantified in the urine. After overnight fasting, participants ingested a beverage containing lactulose (10 g), mannitol (5 g), and sucrose (20 g) within 5 min. They stayed in the laboratory for 4 h and 30 min. A quantity of 150 mL water was administered after 2 and 3 h of testing; no other food or drink was allowed. The total volume of fasting urine collected in a container and postprandial urine was aliquoted and added with thimerosal reagent to restrain microbial growth. The samples were stored at −20 °C until the analysis. For the analysis, the urine samples were unfrozen, homogenized in a vortex, and filtered with a syringe with an attached 30 mm PES membrane filter. Urine samples were dispensed directly to vials to be read in a High-Performance Liquid Chromatography (HPLC) device that informed on the amount of mannitol and lactulose excreted in the urine. The lactulose/mannitol ratio, % lactulose, and % mannitol excreted were calculated for inter- and intra-group comparisons.

Plasma zonulin and lipopolysaccharide-binding protein (LBP) concentrations were measured by enzyme-linked immunosorbent assay (ELISA) using a specific analysis kit (MyBiosource^®^ Human Zonulin, Cat. N° MBS167049, San Diego, CA, USA; HycultBiotech^®^, Wayne, PA, USA; Cat. No. HK315-02). A spectrophotometer (Multiskan™ FC Microplate Photometer, Thermo Scientific™, Waltham, MA, USA) at 450 nm was used for data reading and a standard curve was used to convert data into results, expressed in ng/mL.

SCFA analysis considered 100 mg of fecal samples diluted in 1 mL ultra-pure water, homogenized in vortex, and the supernatant was collected and treated with reagents for assessing short-chain fatty acids. In brief, the feces were thawed at room temperature and aliquoted to be diluted in ultrapure water. The mixture was homogenized without vortexing (5 min). A total of 600 μL supernatant was transferred to a microtube where a calcium hydroxide solution (600 μL) and a cupric sulfate solution (300 μL) were incorporated, followed by homogenization with the vortex for 10 s and freezing. Then, the samples were thawed and centrifuged at 12,000× *g* for 10 min. The supernatant (1.0 mL) was transferred to new microcentrifuge tubes, added with 28 μL of concentrated sulfuric acid, and frozen again. Lastly, the samples thawed and centrifuged at 12,000× *g* for 10 min. A 600 µL aliquot of the supernatant was transferred into HPLC vials for analysis that was performed using HPLC with a Dionex Ultimate 3000 Dual chromatograph (Thermo Scientific, Sunnyvale, CA, USA), coupled to a Shodex RI-101 refractive index detector (Resonac Corporation -Shodex, Tokyo, Japan), which was maintained at 40 °C. The organic acids were separated isocratically on a 300 mm × 7.8 mm Phenomenex Rezex ROA column maintained at 40 °C. The mobile phase employed 5 mM sulfuric acid with a flow rate of 0.7 mL/min. Acetic, propionic, and butyric acids were used as external standards in the calibration curve.

Fecal pH was determined with a PH-1900 model pH meter (Instrutherm^®^, São Paulo, SP, Brazil) after the dilution and homogenization of 1 g of feces in a total of 10 mL of deionized water. Subsequently, the pH glass electrode was completely inserted in the sample and maintained for 1 min to measurement. It was carried out in duplicate [25].

### 2.8. Statistical Analysis

To evaluate the distribution of the variables, the Shapiro–Wilk test, along with histograms and boxplots, were employed. For within-group comparisons between baseline and post-intervention values, a paired *t*-test was applied when variables followed a normal distribution, while the Wilcoxon test was employed for those without a normal distribution. Comparisons between groups used independent *t*-tests (normal distribution) or the Mann–Whitney U test (without normal distribution). Normally distributed variables were expressed as mean (standard deviation) and for non-normally distributed groups as median [interquartile range (25th–75th percentiles)]. For database creation, Microsoft Office Excel version 16.49, 2021^©^ was used and statistical analysis performed with SPSS software (IBM Corp., SPSS Statistics for Windows, Version 25.0, Armonk, NY, USA). A significance level of 0.05 was used for all statistical analyses.

### 2.9. Microbiota Analysis

Genomic DNA was extracted at the Experimental Nutrition Laboratory (UFV, Brazil) using a QIAamp^®^ DNA Mini Kit (Qiagen, Hilden, Germany), following the manufacturer’s protocol. DNA purity was assessed by the A260/A280 ratio through a NanoDrop 7000 Spectrophotometer (Thermo Fisher Scientific, Waltham, MA, USA), while integrity was verified through 1% agarose gel electrophoresis. The amplification of the V4 region of the prokaryotic 16S rRNA gene sequences used Next-Generation Sequencing (NGS) with the universal primers (515F 5′-GTGCCAGCMGCCGCGGTAA-3′ and 806R 5′-GGACTACHVGGGTWTCTAAT-3′) at the Genome Research Core—University of Illinois (Chicago, IL, USA). PCR products were sequenced on an Illumina NovaSeq6000 instrument according to the standard protocols (Illumina, Inc., San Diego, CA, USA).

For 16S sequencing data processing, the software QIIME2 (v. 2020_8) was employed [26]. Paired V4 16S rRNA sequences were processed and trimmed using the cutadapt plugin within QIIME2 [27]. Amplicon sequence variants (ASVs) were extracted using the DADA2 plugin in QIIME2 [28]. To assign taxonomies, a classifier trained on Ribosomal Database Project (RDP) taxonomy training set No. 19 [29] was employed, using the feature-classifier plugin in QIIME2 [30].

Subsequent microbiota analysis was performed using statistical software R 4.3.3. Diversity analysis was performed using the vegan package in R. For alpha diversity, Chao 1 and Shannon indices were calculated and compared by Wilcoxon Rank Sum Test. Beta-diversity was assessed by the Bray–Curtis distance matrix, and adonis2 function (PERMANOVA) was used to test significance. The Bacillota/Bacteroidota (namely Firmicutes/Bacteroidetes) ratio was calculated and compared using the Wilcoxon Rank Sum Test. Differential analysis to find discriminant taxa between groups was performed by transforming counts by the centered log-ratio (CLR) [31] and using generalized linear mixed model implementation by the lmerTest package in R [32] with a random effect for individual variation. Significance was determined by a 0.1 threshold for false discovery rate (FDR)-adjusted *p*-values by Benjamani–Hochberg. The association of changes in microbiota with putative metabolites found only in the KGwas made by Spearman correlation, considering significance at *p* < 0.05.

### 2.10. Untargeted Blood Metabolome

Serum samples obtained from each participant enrolled in this study were subjected to metabolomics analysis at the Metabolomics Unit of the University of Navarra (Spain). Untargeted blood metabolome analysis was performed to assess whether there was any change in the metabolite profile after kombucha consumption. Chromatographic analysis with an HPLC from Agilent (model 1200) coupled to a Time of Flight (TOF) Mass Accuracy device from Agilent (model 6220), operated in positive electrospray ionization mode (ESI+) and negative mode (ESI−) (LC–MS), was used to perform this analysis.

The samples stored at −80 °C were prepared by defrosting and homogenizing. Thus, a total of 150 μL aliquots were pipetted and 450 μL of methanol (MeOH, grade LC–MS, Scharlab, Barcelona, Spain) was added to each aliquot. The samples were vigorously vortexed for 1 min (VX-2500 multi-tube vortexer, VWR, Radnor, PA, USA) and posteriorly centrifuged at 14,756× *g* for 10 min (Biofuge A, Heraeus Sepatech, Germany). A total of 500 μL of supernatant was collected, evaporated under nitrogen flux for 45 min at 40 °C (Turvovap^®^ LV, Biotage, Upssala, Sweden), and recovered in 150 μL of H_2_O: MeOH 95:5 (*V*:*V*). Afterwards, processed samples were analyzed by LC-MS. The chromatographic column used for the stationary phase was the 150 mm × 46 mm Zorbax SB-C18 column with a 5 μm pore size (Agilent Technologies, Santa Clara, CA, USA), and the mobile phase consisted of milliQ with 0.1% formic acid (canal A) and MeOH with 0.1% formic acid (canal B). The gradient elution was 95% A and 5% B (0–5min); 0% A and 100% B (5–20 min); 95% A and 5% B (20–25 min); 95% A and 5% B (25–30 min). The column temperature was 40 °C. The injection volume was 25 μL and the flow rate was 0.5 mL min^−1^. The TOF detector is made of an electrospray ionized source (ESI) and the following conditions were used: gas temperature, 350 °C; drying gas, 10 L min^−1^; nebulizer, 45 psig; capillary voltage, 3500 V; fragmentor, 175 V; and skimmer, 65 V. The instrument was set out to acquire over the *m*/*z* range of 100–2000 with an acquisition rate of 1.03 spectra/s. To evaluate the quality in this analysis, three types of quality control samples (QCs) were used: (i) a column test, (ii) a pool serum prepared by mixing equal volumes from each of the samples, and (iii) a pool-spiked serum prepared by mixing pool serum with L-phenylalanine, citric acid, caffeine, and leucine (Sigma-Aldrich, St. Louis, MO, USA). The analytical methodology has been detailed in prior publications. [33,34]

MassHunter Qualitative Analysis B.06.00 software (Agilent Technologies, Santa Clara, CA, USA) processed chromatograms to ensure quality. Subsequently, to analyze the results, an alignment with XCMS Online (The Scripps Research Institute, Santa Clara, CA, USA) software was carried out according to a 0.2 min retention time and an accurate mass (deviation of 0.005 Da), to show the features (metabolites/compounds) detected from each chromatogram.

Firstly, a pairwise analysis comparing the end-of-intervention and baseline results was performed separately for the CG and KG. Secondly, metaXCMS Online software was used to confront the results of the pairwise analysis of each group to determine the features that were present only in the KG after the treatment through a Venn diagram [35]. The features common in both groups and presented only in the CG were excluded for the metabolite identification since the objective of the analysis was to strictly evaluate the impact of the beverage on the serum metabolome.

Finally, the MetaboAnalyst software (Xia Lab of McGill University, Quebec, QC, Canada) was used to process the feature results through statistical multivariable tools, such as partial least squares regression (PLS-DA). Raw data were normalized by logarithmic transformation followed by auto scaling (mean-centered and divided by the standard deviation of each variable). The volcano plot identified the most relevant metabolites, using a Fold Change (FC) of 1.5 and *p*-value threshold of 0.05. A Variable Importance in Projection (VIP) of the PLS-DA model was considered with values higher than 1. Subsequently, the features were identified through The Metabolomics Workbench (https://www.metabolomicsworkbench.org/, accessed on 14 May of 2024), which contains structures and annotations of biologically relevant metabolites. The adducts used for the search criteria in the positive polarity were [M+H]^+^, [M+H−H_2_O]^+^, and [M+Na]^+^, and for the negative polarity were [M−H]^−^ and [M−H−H_2_O]^−^; both polarities used a 5 mDa mass tolerance window.

## 3. Results

### 3.1. Participants’ Characteristics and Lifestyle Variables

**Figure 2:**
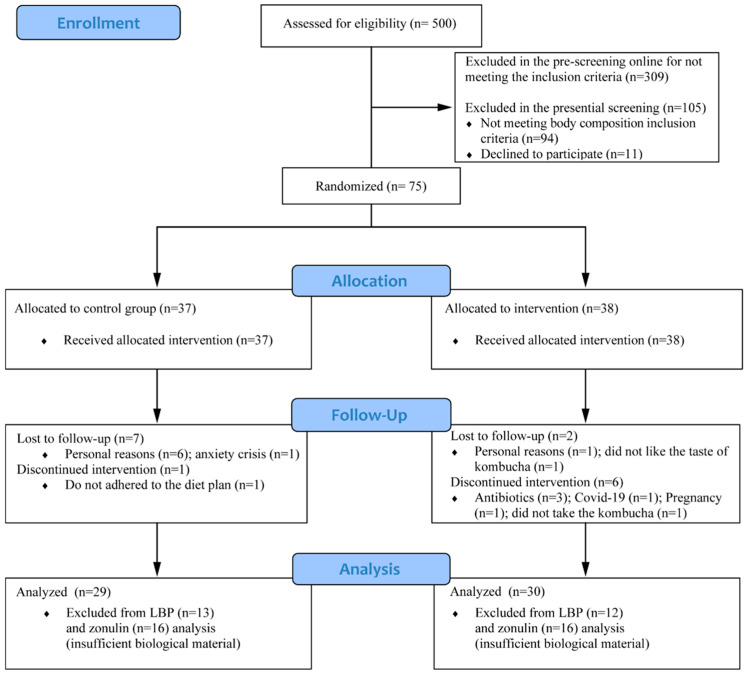
**A** CONSORT flow diagram of the participants. In total, 29 individuals completed the intervention in the control group and 30 in the kombucha group. Analysis considered the totality of participants with exception of LPS-Binding Protein (LBP) and zonulin due to insufficient biological material.

A total of 500 individuals answered the pre-screening form, but 309 did not accomplish the eligibility criteria. After the presential screening at LAMECC, 75 individuals were selected and randomized in the groups: 37 in the CG and 38 in KG (Figure 2). In total, eight individuals in each group discontinued the intervention; as such, 59 finished the intervention, 30 in the KG and 29 in the CG. The exclusions and dropout reasons are described in Figure 2; the data from the participants that did not complete the study were excluded from the statistical analysis.

All individuals had a comparable age—32.5 ± 6.9 years old in the CG and 34.7 ± 6.9 in the KG (*p* = 0.239)—and gender distribution (CG: 17 women/12 men, KG: 18 women/12 men; *p* = 0.914). Both groups initiated the intervention with a similar BMI (CG: 32.34 ± 3.58 kg/m^2^; KG: 33.2 ± 3.7kg/m^2^, *p* = 0.403), WC (CG: 92.8 ± 8.6 cm; KG: 97.2 ± 10.4 cm, *p* = 0.078), and total BF (CG: 45.0 ± 5.9%; KG: 44.7 ± 5.9%, *p* = 0.350) [12]. As presented in previous work, both groups lost weight and almost all the anthropometric and body composition parameters improved, but no differences between the CG and KG were observed [12]. As expected, both groups decreased energy (kcal) consumption, and no differences in macronutrient intake and physical activity level were observed intra- and inter-groups [12]. In this study, we tried to maintain an intake of the usual amount and type of fiber so it would not be considered a bias. Even so, the KG slightly increased their daily fiber intake by 2.9 ± 5.4 as compared to the beginning of the intervention, but this was not statistically significant when compared to the CG (*p* = 0.265) [12].

### 3.2. Quality of Life

After the intervention, the CG total score (*p* = 0.028), physical function (*p* = 0.039), and bodily pain (*p* = 0.002) had increased, as compared with the beginning of the study. In the KG, there was an improvement in the total score (*p* = 0.042), general health (*p* = 0.021), vitality (*p* = 0.045), and role of emotional (*p* = 0.046), comparing the end of the intervention and baseline (Supplementary Material, Figure S1. Changes in Quality of Life through SF-36). However, no differences were found between groups in any of the parameters after the intervention.

### 3.3. Intestinal Parameters and Questionnaires

No significant difference in baseline values between groups were found (zonulin, *p*: 0.108; LBP, *p*: 0.558; butyric acid, *p*: 0.864; acetic acid, *p*: 0.844; propionic acid, *p*: 0.748; fecal pH: 0.142; mannitol excretion, *p*: 0.315; lactulose excretion, *p*: 0.803; L/M ratio, *p*: 0.919).

No differences related to intestinal proteins, zonulin, and LBP, were found between groups after the intervention (zonulin: *p* = 0.928; LBP, *p* = 0.935) or with fecal pH (*p* = 0.296), the excretion (%) of mannitol (*p* = 0.451) and lactulose (*p* = 0.292), and L/M ratio (*p* = 0.938). However, only the CG significantly increased the L/M ratio (*p* = 0.019), fecal pH (*p* = 0.004), and zonulin (*p* = 0.031), compared to baseline values (Table 1). Regarding SCFAs, both the CG and KG decreased the production of butyric acid when comparing baseline with final values across the same group (CG: *p* = 0.044; KG: *p* = 0.020). No other changes were observed regarding SCFAs (Table 1).

In relation to the frequency distribution of the BSS, participants from both groups in baseline and end-of-intervention scores presented a clear predominance in Bristol type 3 and 4; these types together with type 5 are generally considered normal forms (Blake, Raker, Whelan, 2016). In the KG, the type 4 peak at the end of intervention was 16.67% higher compared to the baseline value. Moreover, individuals who consumed GTK did not report abnormally hard (type 1 and 2) or loose/liquids stools (type 6 and 7). In contrast, in the CG, 6.9% participants (*n* = 2) mentioned abnormal stool consistency after intervention, with one registering type 1 and one type 6. Nevertheless, the majority of individuals in the CG mentioned a normal BSS score (Supplementary Material, Figure S2. Frequency Distribution of Bristol Consistency Scale).

**Figure 3:**
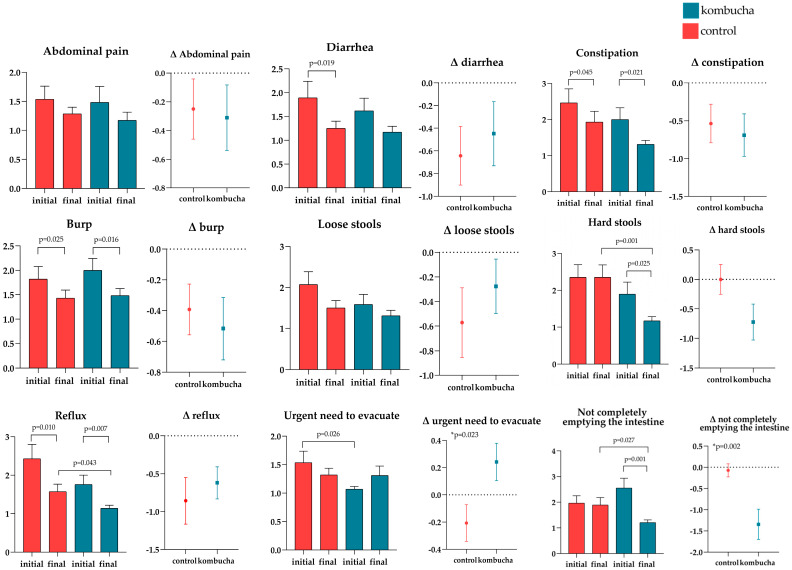

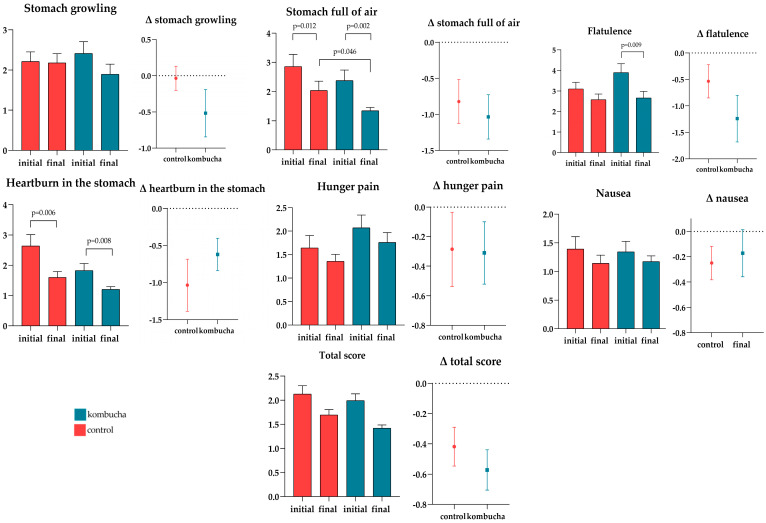
Comparison of gastrointestinal symptoms evaluated through the Gastrointestinal Symptom Rating Scale (GSRS) questionnaire, according to the allocation group. Values expressed as means (SEM). Comparison between baseline and endpoint results across the same group (paired *t*-test) and comparisons between baseline, endpoint and Δ between groups (independent *t*-test), only significant *p*-values expressed (<0.05). Δ = final *−* baseline.

Although the individuals did not show major changes in the BSS, improvements in gastrointestinal symptoms through GSRS were reported in the CG and KG (Figure 3). Both groups mentioned a decrease in several gastrointestinal symptoms, such as heartburn in the stomach and burping, when comparing baseline with end-of-intervention data. Between-group comparison demonstrated that after the intervention, the KG presented lower values for total score (*p* = 0.035), reflux (*p* = 0.043), having a stomach full of air (*p* = 0.046), hard stools (*p* = 0.001), and not completely emptying the intestine (*p* = 0.027). Moreover, the KG decreased more regarding the symptom of not completely emptying the intestine when compared to the CG (ΔKG: −1.34 ± 1.91 vs. ΔCG: −0.07 ± 0.81, *p* = 0.002). The CG reported a significant decrease of having an urgent need to evacuate compared to the KG (ΔKG: 0.24 ±0.74 vs. ΔCG: −0.21 ± 0.73, *p* = 0.032), but at baseline the inter-group values were already different (KG: 1.07 ± 0.25 vs. CG: 1.54 ± 1.07, *p* = 0.026) (Figure 3).

### 3.4. Fecal Microbiota

Differential abundance analysis between the two groups showed changes in family, genus, and species levels, but when the *p*-values were adjusted by Benjamani–Hochberg, no statistical significance was observed (Table 2). At the genus level, for example, Alistipes Odoribacter and Parabacteroides presented a tendency to decrease and Romboutsia to increase in the KG (Table 2). No differences in the Bacillota/Bacteroidota ratio were observed from baseline and endpoint in the CG (*p* = 0.80) and KG (*p* = 0.58) or between groups after the intervention (*p* = 0.38).

**Figure 4:**
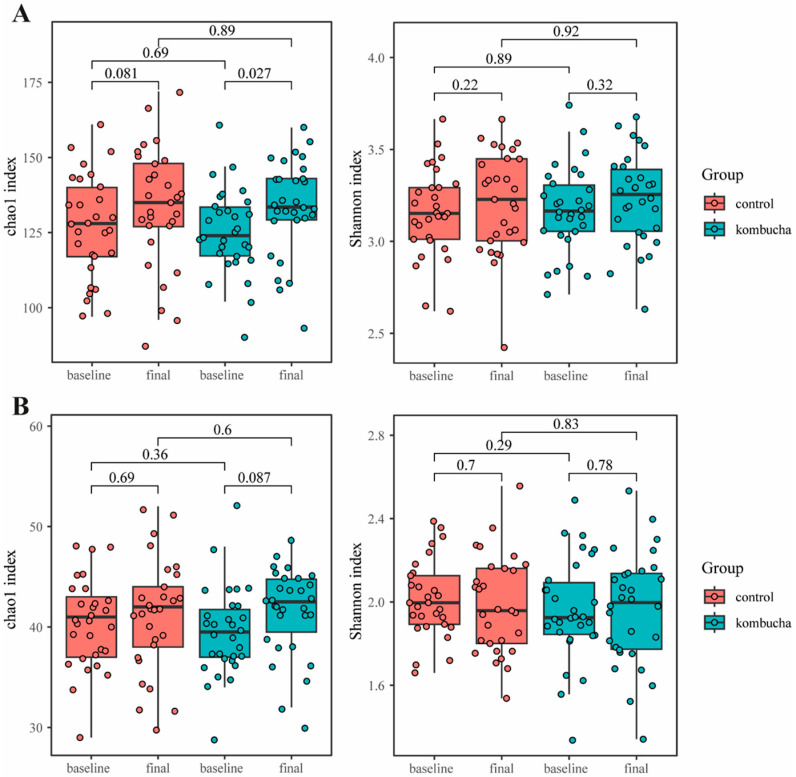
Alpha diversity represented by Chao 1 and Shannon indices according to allocation group (control: red; kombucha: blue) and intervention visit (baseline and final). (**A**) Chao 1 and Shannon indices by genus level and (**B**) by family level. Values were compared by Wilcoxon Rank Sum Test.

The alpha diversity measured by Chao 1 at the ASV and species level was higher both in the CG (*p* = 0.014; *p* = 0.027, respectively) and KG (*p* = 0.036; *p* = 0.013, respectively) when comparing the beginning with the end of study, but no differences between groups were found (*p* = 0.95; *p* = 0.74, respectively). At the genus level, Chao 1 increased only in the KG (*p* = 0.027) when compared to the baseline, but no differences were found compared to the CG after the intervention (*p* = 0.89) (Figure 4). The Shannon index did not change after the intervention (Figure 4). Moreover, beta diversity analysis by Bray–Curtis dissimilarity did not present differences between groups at any level after the intervention (*p* > 0.05).

### 3.5. Serum Metabolomics

**Figure 5:**
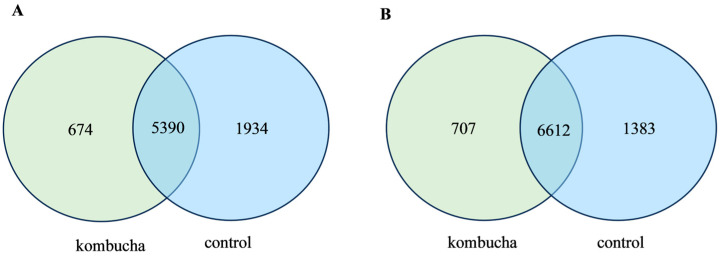
Venn diagram in ESI+ (**A**) and ESI− (**B**) modes showing metabolites common in both groups and those detected in just kombucha and control groups.

**Figure 6:**
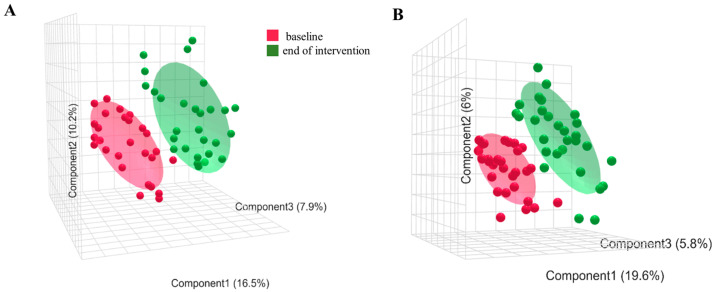
PLS-DA plots representing the baseline (color: red) and end-of-intervention (color: green) data of the kombucha group in ESI+ (**A**) and ESI− (**B**).

The HPLC-TOF-MS method detected 7998 (ESI+ mode) and 8702 features (ESI− mode) using MetaXCMS-analysis. From the total features detected in the ESI+ mode, 674 were found just in the KG, 1934 just in the CG, and 5390 were common in both groups (Figure 5). In respect to the features of the ESI- mode, 6612 were common in both groups, 1383 were present in just the CG, and 707 in just the KG (Figure 5). As such, only the discriminant features detected in the KG in the positive and negative polarity were used for identification, since this study aimed to assess the changes that the beverage caused in the serum metabolome. In the PLS-DA, it is possible to see a separation between pre- and post-intervention values among the KG in both polarities (Figure 6).

Only the features that VIP > 1 were considered as discriminant metabolites. This resulted in a total of 25 putative metabolites detected in ESI+ mode after the consumption of GTK, and 30 putative metabolites in ESI− mode. After exclusion of drugs/toxic, 14 metabolites remained in ESI+ and 15 in ESI− mode. In many instances, the discriminating compounds identified exhibited several potential putative metabolites. Existing literature was assessed to choose the most probable metabolites according to the primary outcome of this intervention, allowing for the selection of the most suitable markers. Most of the relevant putative metabolites detected after GTK consumption were compounds derived from fungi. The other metabolites found were related to amino acids and fatty acid metabolism, derived from plants and components such as vitamins and phenolic compound (Table 3).

**Figure 7:**
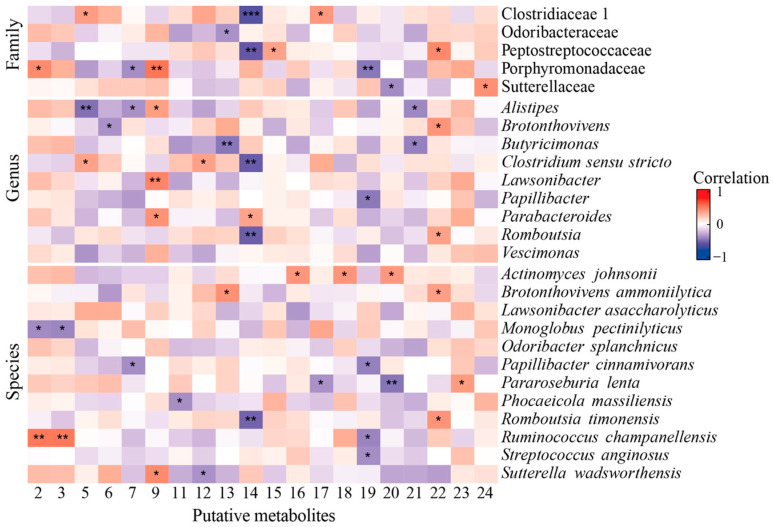
Correlation chart for changes in microbiota and putative metabolites found only in kombucha group after intervention. Sperman correlation considered with significance at *p* < 0.05. Red indicates positive correlation and purple negative correlation. ***: FDR < 0.001; **: FDR < 0.01; *: *p* < 0.05.

Figure 7 shows the correlations between changes in microbiota and putative metabolites presented in the KG after the intervention. For example, Beta-D-Glucopyranosyl4-O-Beta-D-Glucopyranosyl-beta-D-glucopyranoside and 5-L-Glutamyl-L-alanine were negatively associated with the decreased abundance of the genus *Alistipes*. Moreover, pantothenamide showed a positive correlation with lower Sutterellaceae levels, and taurine a negative correlation with a higher abundance of the genus *Romboutsia* and families Clostridiaceae 1 and Peptotostreptococcaceae. Furthermore, diethyl malonate showed a positive correlation with *Romboutsia* (Figure 7).

## 4. Discussion

Kombucha consumption has increased in recent years because of its promising health benefits, such as its probiotic, antioxidant, anti-inflammatory, and anti-obesity [2,36,37] benefits. These effects have been confirmed in vitro and in animal models, but to our knowledge, very few trials have investigated its impact on human health [37,38,39,40]. Corroborating the results of this intervention, Isakov et al. (2023) demonstrated that individuals who consumed kombucha significantly reported an improvement in gastrointestinal symptoms, mainly decreasing the severity of incomplete bowel emptying. The authors raised the hypothesis that the organic acids normally presented in kombucha, such as acetic, citric, and malic acid, may be responsible for the increased motility. First, the increased secretion of prostaglandin E2 binds to specific receptors on intestinal muscle cells, such as EP3 receptors, which might enhance the frequency and amplitude of peristaltic contractions. Secondly, the decreased expression of aquaporin 3 inhibits water reabsorption from the intestinal lumen, which might increase stool volume and reduce hardness [40,41]. Indeed, in our results, GTK appears to stimulate gastrointestinal transit, since the KG also decreased as regards the symptoms of not completely emptying the intestine and hard stools. In this manner, it may be a good strategy to be prescribe GTK in the prevention of constipation and irregular stool motility that is usually present in individuals with excess body weight [42].

Kombucha has been suggested to improve damaged intestinal barriers caused by systemic low-grade inflammation, by decreasing serum LPS and increasing the expression levels of tight junction proteins (zonulin, claudin, and occludin) and mucin-related genes in globet cells [43]. In our results, GTK did not impact plasma zonulin and other intestinal permeability markers; however, only the CG presented a worsening in zonulin and the L/M ratio. These data suggest that somehow the GTK neutralized the worsening of these variables in the individuals who consumed the beverage. Since the integrity of the intestinal barrier is associated with systemic inflammation [43], this result in the CG may be related to the significant increase in IL-6 also observed only in this group, described previously in [12], possibly reflecting the physiological adaptations of energy allocation and lipid mobilization present in caloric restriction and weight loss. Indeed, higher serum/plasma zonulin levels have been associated with higher inflammatory markers, including IL-6 [44,45].

Moreover, fermentation modifies the tea composition due to the degradation of complex substances that results in the increased concentration of total phenolic compounds [46]. These phenolic compounds can act as prebiotics since only 5–10% is absorbed in the small intestine and sequentially reaches the colon to undergo a biotransformation by the colonic microbiota [47,48]. The metabolization of phenolic compounds may stimulate the production of SCFA, decrease fecal pH, and, consequently, improve intestinal permeability and protect against pathogenic bacteria through antimicrobial activity [43,49]. In fact, three studies in animal models showed that GTK promoted the growth of SCFA-producing bacteria in mice with LPS-induced sepsis [50], type 2 diabetes mellitus [43], and in Wistar rats fed a high-fat high-fructose diet [10]. In our study, GTK consumption did not enhance the production of SCFAs. One possible explanation may lie in the concentration of phenolic compounds present. After five days of fermentation, GTK contained 64 mg of total phenolics per 200 mL, primarily flavonoids such as epigallocatechin 3-O-gallate and epicatechin [12]. Although these compounds have potential health benefits, their concentration may have been insufficient to stimulate notable SCFA production. Furthermore, the specific microbial community composition and metabolic pathways in the gut of the study participants might also influence SCFA production. Thus, while GTK provides bioactive compounds, it may not reach the threshold of the phenolic compound concentration or specific microbial modulation necessary to significantly enhance SCFA production.

The final enriched composition of GTK, together with its important microbial profile, may aggregate the potential microbiota modulatory effect of the drink [43]. Contrariwise, the KG did not present a significant change in microbiota abundance or diversity compared with the CG, after *p*-value adjustment (FDR). However, relevant tendencies in microbiota composition were found after GTK intake, such as the decreased abundance of *Sutterella wadsworthensis,* which has been previously positively correlated with obesity [51] and vascular stiffness [52], and the higher abundance of *Roseburia intestinalis*, which plays an important role in regulating intestinal barrier homeostasis, preventing inflammation, and maintaining energy homeostasis [53]. Furthermore, the lower abundance of *Parabacteroides* is noteworthy, since this genus has been associated with non-alcoholic fat liver disease [54], and the higher abundance of *Blautia obeum* is also noteworthy, which has been related to anti-proliferative properties, as with, for example, the disabling of the pathogenic mechanism of *Vibrio cholerae* [55].

On the other hand, a worsening tendency has been observed regarding some genera, such as the decreased abundance of *Odoribacter,* which has been related to different microbiota-associated diseases, like non-alcoholic fatty liver disease, cystic fibrosis, and inflammatory bowel disease [56]. Also, the lower abundance of *Alistipes* has been negatively correlated with body weight, lipid profile, blood glucose, and pressure in a study that assessed the gut microbiota of 1914 adults for the classification of obesity-related metabolic abnormalities [57].

Regarding the metabolomics analysis, we only found discriminant metabolites present in the KG after the intervention, such as diethyl malonate, which is a compound produced from *Saccharomyces cerevisiae*, one of the main yeast strains commonly present in kombucha [57,58]. Diethyl malonate isolated from kombucha tea has shown cytotoxic and anti-invasive properties against cancer cell lines [59]. This compound was positively correlated with the increase in *Romboutsia* presented in the KG after the intervention. The increased abundance of this genus has also been found after treatment with other fermented tea (heimao tea) and it was negatively correlated with body weight and positively correlated with the interscapular brown adipose tissue [60].

Taurine also increased in the serum metabolome after GTK consumption. It is involved in bile acid conjugation, antioxidant and anti-inflammatory effects beyond the impact of the intestinal microbiota, and a reduced risk of metabolic syndrome [61,62,63]. Kombucha has a composition rich in several chemical components, such as organic acids, complex B and C vitamins, enzymes, and free amino acids (AA) [64]. Among the AA, methionine can be present in the beverage, which together with cysteine is one of the essential components needed to synthesize taurine [64]. Moreover, the production of gluconic, glucuronic, and D-saccharic-1,4-lactone organic acids after fermentation confers to the drink a hepatoprotective effect that could enhance liver health and consequently improve its capacity to synthesize taurine [62,65]. Taurine has been negatively correlated with the increased abundance of pathogens like the *Clostridium sensu stricto* genus and Peptostreptococcaceae family, this different from other studies that observed a positive correlation [66,67]. Although neither study evaluated taurine in the serum metabolome of humans, Zhang et al. (2018) analyzed the content of taurine in luminal and mucosal tissues from the ileum, cecum, and colon of pigs [66], and Wang et al. (2023) studied the effect of taurine supplementation in early weaned piglets [67].

Among the metabolites derived from medicinal plants, we found asperuloside, which is an iridoid glycoside secondary metabolite. This compound has been related to anti-obesity, anti-inflammatory, and antioxidant effects via the inhibition of the NF-κB pathway and consequent lowering of the secretion of inflammatory cytokines and reactive oxygen species [68]. Furthermore, compounds derived from fungi were also present after GTK intake, as with, for example, cytosporone, which is a polyketide-derived octaketide phenolic lipid reported to have antimicrobial, antimalarial, cytotoxic, antivirus, and anti-inflammatory activities [69,70].

One notable strength of this study is the implementation of a meticulously randomized controlled trial with a duration of 10 weeks. This is a pioneering study evaluating the impact of GTK on intestinal health and serum metabolome in humans in weight loss treatment. Since several lifestyle factors impact the microbiota, we tried to control various confounders such as alcohol consumption, medication, smoking, dietary patterns and fiber intake, age, sex, and initial body composition. However, our study has limitations such as a relatively small sample size and the lower number of samples for the LBP and zonulin analysis.

## 5. Conclusions

Overall, green tea kombucha enhanced the improvement in gastrointestinal symptoms in individuals with excess body weight undergoing weight loss treatment, mainly helping in the sensation of completely emptying the intestine and hard stools, important for the optimal function and motility of the intestine. Moreover, although green tea kombucha did not modulate gut microbiota after *p*-value adjustment, it demonstrated some positive tendencies that should be confirmed in other clinical trials involving individuals with excess body weight. Another important result was the worsening of intestinal permeability markers and fecal pH only in the control group, suggesting that kombucha intake might partially prevent these negative effects.

Finally, green tea kombucha consumption impacted the serum metabolome. Some of the metabolites found strictly in the kombucha group are related to anti-obesity, anti-inflammatory, and antioxidant properties that may improve the overall health of individuals with excess body weight.

## Figures and Tables

**Table 1:** Comparison of intestinal markers, according to allocation groups.

	CG (*n* = 29)				KG (*n* = 30)			
	Baseline	After 10 Weeks	Δ	* *p*-Value	Baseline	After 10 Weeks	Δ	* *p*-Value
Mannitol excretion (%)	16.5 (6.6)	12.0 (5.7)	−4.5 (7.62)	**0.005**	15.1 (3.9)	13.1 (3.9)	−2.0 (4.59)	**0.024**
Lactulose excretion (%)	0.24 (0.12)	0.25 (0.13)	0.01 (0.15)	0.885	0.23 (0.11)	0.38 (0.60)	0.14 (0.64)	0.202
L/M ratio	0.015 (0.007)	0.028 (0.025)	0.012 (0.026)	**0.019**	0.016 (0.006)	0.028 (0.030)	0.012 (0.032)	0.060
Fecal pH	7.3 (0.7)	7.7 (0.6)	0.4 (0.7)	**0.004**	7.6 (0.7)	7.5 (0.5)	−0.04 (0.7)	0.711
Butyric acid (mmol/L)	5.8 (3.4–6.9)	2.8 (1.6–5.2)	−2.1 (−4.1–1.0)	**0.044**	5.6 (3.5–6.8)	3.2 (2.5–5.0)	−2.0 (−3.9–0.7)	**0.020**
Acetic acid (mmol/L)	16.3 (10.1–23.8)	13.6 (7.1–27.9)	−2.9 (−10.3–8.7)	0.633	16.4 (11.6–24.3)	16.6 (10.2–21.2)	−1.5 (−9.8–7.8)	0.581
Propionic acid (mmol/L)	7.1 (4.5–10.4)	5.8 (3.1–9.0)	−1.4 (−4.3–3.6)	0.285	6.7 (5.1–10.5)	6.1 (4.1–8.9)	−1.5 (−6.3–3.3)	0.316
LBP ^a^ (ng/mL)	14.3 (3.5)	13.6 (3.7)	−0.6 (3.5)	0.487	15.2 (5.6)	14.5 (4.2)	−0.7 (3.6)	0.398
Zonulin ^b^ (ng/mL)	17.9 (14.5–24.7)	20.5 (16.9–26.8)	2.5 (−0.3–5.0)	**0.031**	20.2 (14.2–26.9)	20.5 (16.3–33.4)	0.3 (−1.42–8.0)	0.316

Quantitative data expressed as mean (SD) or median (percentile 25–75). * *p*-value: comparison between baseline and endpoint results across same group (paired *t*-test or Wilcoxon). Values with statistically significant difference indicated in bold. No significant differences in baseline or final values between groups were found (independent *t*-test or Mann–Whitney test). ^a^: CG: *n* = 13; KG: *n* = 14. ^b^: CG: *n* = 16; KG: *n* = 18. CG: control group; KG: kombucha group. LBP: lipopolysaccharide binding protein; L/M: lactulose/mannitol ratio; Δ = final − baseline.

**Table 2:** Bacterial taxa statistically significant between groups after intervention.

		Effect	*p*-Values	FDR	↓/↑Kombucha
Family	Odoribacteraceae	−1.2083	0.00781	0.39355	↓
	Clostridiaceae	1.5735	0.01543	0.39355	↑
	Sutterellaceae	−0.985	0.02669	0.45377	↓
	Rikenellaceae	−0.678	0.04756	0.48216	↓
	Porphyromonadaceae	−0.6163	0.05017	0.48216	↓
	Lactobacillaceae	1.40983	0.07003	0.48216	↑
	Peptostreptococcaceae	0.56863	0.07254	0.48216	↑
	Acidaminococcaceae	−0.7804	0.07563	0.48216	↓
Genus	*Clostridium sensu stricto*	1.5748	0.01455	0.96936	↑
	*Romboutsia*	1.09737	0.0167	0.96936	↑
	*Brotonthovivens*	1.05662	0.02389	0.96936	↑
	*Odoribacter*	−0.8116	0.03586	0.96936	↓
	*Alistipes*	−0.6215	0.05056	0.96936	↓
	*Papillibacter*	−0.8804	0.05471	0.96936	↓
	*Vescimonas*	−0.4248	0.06337	0.96936	↓
	*Butyricimonas*	−1.0629	0.06871	0.96936	↓
	*Parabacteroides*	−0.5285	0.09511	0.96936	↓
	*Lawsonibacter*	−0.6426	0.0993	0.96936	↓
Species	*Romboutsia timonensis*	1.12236	0.01714	0.99383	↑
	*Lawsonibacter asaccharolyticus*	−0.9777	0.01748	0.99383	↓
	*Brotonthovivens ammoniilytica*	1.09341	0.01834	0.99383	↑
	*Sutterella wadsworthensis*	−0.8522	0.02095	0.99383	↓
	*Phocaeicola massiliensis*	−1.0229	0.02253	0.99383	↓
	*Mogibacterium pumilum*	1.13711	0.02733	0.99383	↑
	*Odoribacter splanchnicus*	−0.7748	0.03857	0.99383	↓
	*Actinomyces johnsonii*	0.98591	0.04154	0.99383	↑
	*Ruminococcus champanellensis*	−1.2986	0.05483	0.99383	↓
	*Papillibacter cinnamivorans*	−0.8436	0.06748	0.99383	↓
	*Roseburia intestinalis*	1.23297	0.07291	0.99383	↑
	*Streptococcus anginosus*	0.82715	0.07505	0.99383	↑
	*Blautia obeum*	0.35682	0.07608	0.99383	↑
	*Pararoseburia lenta*	0.35331	0.08584	0.99383	↑
	*Monoglobus pectinilyticus*	0.83951	0.09807	0.99383	↑

Differential analysis was performed using generalized linear mixed models by the lmerTest package in R, adjusting for age and sex as covariates and with a random effect for individual variation. FDR cutoff point < 0.1. ↑: increased abundance in the kombucha group. ↓: decreased abundance in the kombucha group.

**Table 3:** Putative metabolites detected by HPLC-TOF-MS in positive and negative polarity only in the kombucha group after the intervention.

ID	*m*/*z* (Da)	rt (min)	VIP	Δ (mDa)	Assignment	Putative Metabolite
1	278.1913	20.20	4.78	0.1	[M−H−H_2_O]^−^	hexadeca-2E,4E,9Z-triene-12,14-diynoic acid iso…
2	325.1657	9.00	4.42	1.2	[M+H]^+^	Cytosporone M
3	311.1952	8.99	3.76	3.0	[M+Na]^+^	Flavodonfuran
4	139.0085	16.27	3.37	4.8	[M−H−H_2_O]^−^	Maleylacetic acid
5	395.0973	24.76	3.10	1	[M−H−H_2_O]^−^	Asperuloside
6	416.1231	25.27	2.99	2	[M−H−H_2_O]^−^	Terremide D
7	443.1242	7.87	2.93	4.7	[M−H]^−^	Beta-D-Glucopyranosyl 4-O-Beta-D-Glucopyranosyl-beta-D-glucopyranoside
8	357.1204	16.91	2.83	0.1	[M+Na]^+^	tripeptide Ala-Asn-Met
	357.1204	16.91	2.83	0.1	[M+Na]^+^	tripeptide Asn-Cys-Val
	357.1204	16.91	2.83	0.1	[M+Na]^+^	tripeptide Gln-Gly-Met
9	415.1263	17.10	2.74	0.4	[M−H−H_2_O]^−^	tripeptide Asp-Asp-Trp
10	627.4813	25.66	2.67	0.6	[M+Na]^+^	PE-Cer(d14:2(4E,6E)/19:0)
11	307.2849	24.67	2.61	0.7	[M+H−H_2_O]^+^	Hexahydropolyandrocarpidine
12	369.2062	24.73	2.60	0.1	[M+H−H_2_O]^+^	Ankaflavin
13	367.2228	23.05	2.49	1.6	[M+Na]^+^	3-Hydroxyneogrifolin
	367.2228	23.05	2.49	1.6	[M+Na]^+^	Abiesadine P
14	124.0089	9.10	2.38	1.5	[M−H]^−^	Taurine
15	431.2239	14.46	2.30	1.1	[M−H]^−^	Gombapyrone F
	431.2239	14.46	2.30	1.1	[M−H−H_2_O]^−^	Azadiradione
16	505.3841	23.70	2.20	4.6	[M+H]^+^	Ganoderiol G
17	124.9531	13.14	2.00	1.7	[M+H]^+^	1,2,4-Trithiolane
18	246.0951	8.66	1.98	2.1	[M+H]^+^	Mycosporine glycine
19	327.1312	14.69	1.92	0.2	[M−H−H_2_O]^−^	tripeptide Ala-Glu-Gln
	327.1312	14.69	1.92	0.2	[M−H−H_2_O]^−^	tripeptide Asn-Asp-Val
20	1143.808	19.28	1.89	4.5	[M−H−H_2_O]^−^	Trichoderin A
21	199.069	8.33	1.84	3.4	[M−H−H_2_O]^−^	5-L-Glutamyl-L-alanine
22	183.0589	16.69	1.68	3.9	[M+Na]^+^	Diethyl malonate
23	124.0166	17.09	1.63	0.3	[M−H]^−^	L-(1-aminoethyl)phosphonic acid
24	217.1188	10.40	1.58	0.6	[M−H]^−^	Pantothenamide

## Data Availability

The data supporting the results of this study can be obtained by contacting the corresponding author. These data are not publicly accessible as they include personal information that may affect the privacy of the participants.

## Supplementary Materials

The following supporting information can be downloaded at https://www.mdpi.com/article/10.3390/foods13223635/s1, Figure S1: Changes in Quality of Life through the SF-36 questionnaire; Figure S2: Frequency Distribution of Bristol Stool Scale.
