# Supplementary material for: The Impact of Green Tea Kombucha on the Intestinal Health, Gut Microbiota, and Serum Metabolome of Individuals with Excess Body Weight in a Weight Loss Intervention: A Randomized Controlled Trial
Source: Foods. 2024 Nov 14;13(22):3635. doi: 10.3390/foods13223635 (PMC11594279; doi:10.3390/foods13223635)
Supplement: Supplementary file 1 [file foods-13-03635-s001.zip › Supplementary Material.pdf]

Supplementary Material

Figure S1. Changes in Quality of Life through the SF-36 questionnaire.

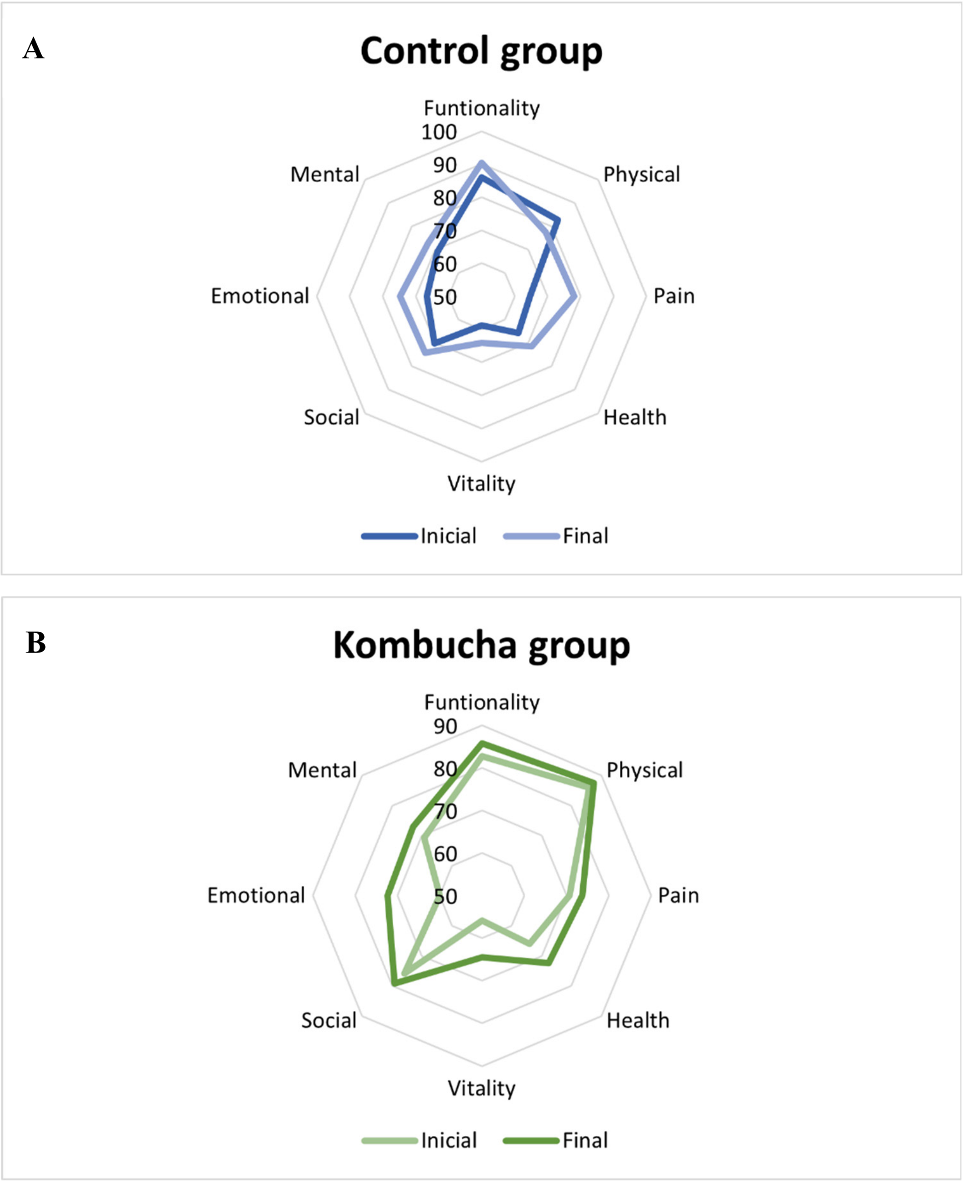

Figure S1. Radar charts representing changes in quality-of-life domains by 36-Item Short Form Health Survey (SF-36) comparing final of intervention with baseline in (A) control group and (B) kombucha group.

Figure S2. Frequency Distribution of Bristol Stool Scale.

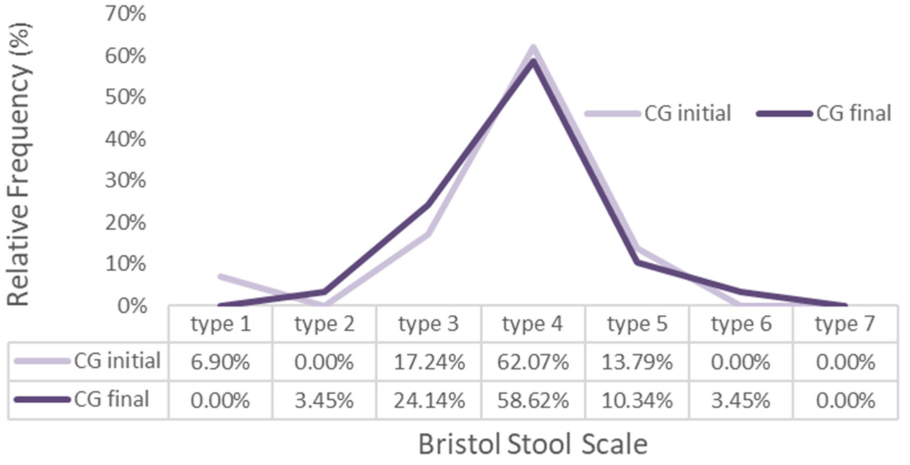

	type 1	type 2	type 3	type 4	type 5	type 6	type 7
CG initial	6.90%	0.00%	17.24%	62.07%	13.79%	0.00%	0.00%
CG final	0.00%	3.45%	24.14%	58.62%	10.34%	3.45%	0.00%

A

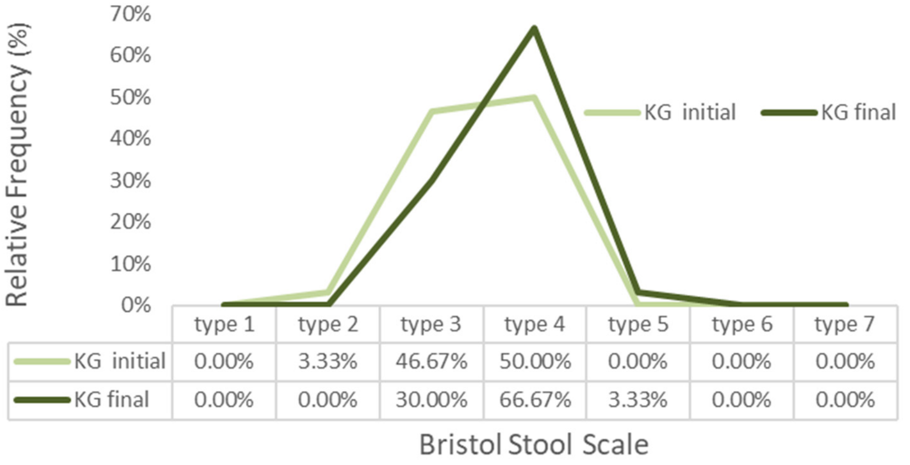

	type 1	type 2	type 3	type 4	type 5	type 6	type 7
KG initial	0.00%	3.33%	46.67%	50.00%	0.00%	0.00%	0.00%
KG final	0.00%	0.00%	30.00%	66.67%	3.33%	0.00%	0.00%

B

Figure S2. Relative frequency distribution of Bristol Stool Scale (BSS) in the baseline and end of intervention in (A) control group and (B) kombucha group.
